# Glacial changes in sea level modulated millennial-scale variability of Southeast Asian autumn monsoon rainfall

**DOI:** 10.1073/pnas.2219489120

**Published:** 2023-06-26

**Authors:** Elizabeth W. Patterson, Kathleen R. Johnson, Michael L. Griffiths, Christopher W. Kinsley, David McGee, Xiaojing Du, Tamara Pico, Annabel Wolf, Vasile Ersek, Richard A. Mortlock, Kweku A. Yamoah, Thành N. Bùi, Mùi X. Trần, Quốc Đỗ-Trọng, Trí V. Võ, Trí H. Đinh

**Affiliations:** ^a^Department of Earth System Science, University of California, Irvine, CA 92617; ^b^Department of Environmental Science, William Paterson University, Wayne, NJ 07470; ^c^Berkeley Geochronology Center, Berkeley, CA 94709; ^d^Department of Earth, Atmospheric and Planetary Sciences, Massachusetts Institute of Technology, Cambridge, MA 02139; ^e^Department of Earth, Environmental, and Planetary Sciences, Brown University, Providence, RI 02912; ^f^Department Earth and Planetary Sciences, University of California, Santa Cruz, CA 95064; ^g^Department of Geography and Environmental Sciences, Northumbria University, Newcastle NE1 8ST, United Kingdom; ^h^Department Earth and Planetary Sciences, Rutgers University, Piscataway, NJ 08854; ^i^Department of Archaeology, BioArc, University of York, York YO10 5DD, United Kingdom; ^j^Phong Nha-Ke Bang National Park, Phong Nha 510000, Vietnam; ^k^University of Science, Vietnam National University, Hanoi 100000, Vietnam

**Keywords:** Vietnam, paleoclimate, autumn monsoon, speleothem, sea level

## Abstract

Most paleoclimate studies of Mainland Southeast Asia hydroclimate focus on the summer monsoon, with few studies investigating rainfall in other seasons. Here, we present a multiproxy stalagmite record (45,000 to 4,000 years) from central Vietnam, a region that receives most of its annual rainfall in autumn (September-November). We find evidence of a prolonged dry period spanning the last glacial maximum that is punctuated by an abrupt shift to wetter conditions during the deglaciation at ~14 ka. Paired with climate model simulations, we show that sea-level change drives autumn monsoon rainfall variability on glacial-orbital timescales. Consistent with the dry signal in the stalagmite record, climate model simulations reveal that lower glacial sea level exposes land in the Gulf of Tonkin and along the South China Shelf, reducing convection and moisture delivery to central Vietnam. When sea level rises and these landmasses flood at ~14 ka, moisture delivery to central Vietnam increases, causing an abrupt shift from dry to wet conditions. On millennial timescales, we find signatures of well-known Heinrich Stadials (HS) (dry conditions) and Dansgaard–Oeschger Events (wet conditions). Model simulations show that during the dry HS, changes in sea surface temperature related to meltwater forcing cause the formation of an anomalous anticyclone in the Western Pacific, which advects dry air across central Vietnam, decreasing autumn rainfall. Notably, sea level modulates the magnitude of millennial-scale dry and wet phases by muting dry events and enhancing wet events during periods of low sea level, highlighting the importance of this mechanism to autumn monsoon variability.

## Significance

Mainland Southeast Asia is a densely populated region largely devoid of long paleoclimate records. Here, we use a stalagmite from Vietnam to reconstruct Southeast Asian autumn monsoon rainfall variability over the last 45,000 y. We show that sea level, specifically the inundation history of the Gulf of Tonkin and South China Shelf, drove substantial changes in local rainfall. Additionally, we find that autumn rainfall responds to millennial-scale changes of the Atlantic Meridional Overturning Circulation. These findings reveal the considerable variability of the Southeast Asian autumn monsoon, including a propensity for abrupt change. Finally, agreement between our stalagmite record and climate model simulations demonstrates that models correctly capture the main drivers of Southeast Asian autumn monsoon rainfall on millennial-orbital timescales.

The seasonal rains of the Southeast Asian monsoon affect over 650 million people. Relatively small fluctuations in rainfall cause flooding events and prolonged droughts, phenomena that have been affecting the people of Mainland Southeast Asia (MSEA—Vietnam, Laos, Cambodia, Thailand, Myanmar, and Peninsular Malaysia) for millennia (1–3). Paleoclimate records not only reveal these fluctuations in the Southeast Asian monsoon, but also help constrain the dominant drivers of hydroclimate over a range of timescales (1–5). Yet, with few paleorecords from MSEA, the hydroclimate variability of the region remains poorly constrained. Extending the MSEA paleorecord back into the Late Pleistocene will reveal how the regional hydroclimate responded to climate forcings and feedbacks during periods of rapid global change, such as events associated with the last glacial period and deglaciation [e.g., Heinrich Stadials (HS) and Dansgaard–Oeschger (DO) events]. Additionally, ice volume–driven sea-level change impacted MSEA hydroclimate during this time. For example, the relative exposure or inundation of the Sunda and Sahul shelves influenced hydroclimate patterns across MSEA via changes in deep convection in the tropical Indo-Pacific and the resulting reorganization of the Walker Circulation (6–10). We know far less about the effects of land exposure change in northeast MSEA and South China. Even though these landmasses are much smaller than the Sunda and Sahul shelves, their proximal location to MSEA could cause substantial local hydroclimate change. Ultimately, new paleorecords from MSEA serve as crucial benchmarks to test whether the climate models we use to project future climate accurately capture changes in MSEA hydroclimate after large perturbations in external forcings.

The Southeast Asian monsoon, a part of the greater Asian monsoon system, is comprised of two main components, the southwest (SW) monsoon (May-August) with moisture sourced from the Indian Ocean and Bay of Bengal, and the northeast (NE) monsoon (September-April) with moisture sourced from the South China Sea (*SI Appendix*, Figs. S1 *A* and *B* and S2) (11). In contrast to most of MSEA, which receives the majority of its annual rainfall from the SW monsoon during the summer months (July-August; *SI Appendix*, Fig. S1*C*), much of the eastern coast of Vietnam receives a large proportion of its annual rainfall from the NE monsoon during autumn (September-November; *SI Appendix*, Fig. S1*D*); from here on, we refer to this as the Southeast Asian autumn monsoon. While not a common rainfall pattern in the region, the Southeast Asian autumn monsoon is part of the global network of autumn monsoon regions, which occur along the eastern coasts of some landmasses in the tropics (12, 13). During the autumn monsoon, the Truong Son Mountains, a N–S trending mountain range along the Vietnam–Laos border, cause orographic uplift of the NE monsoon winds, which concentrates rainfall on the eastern side of the mountains (14). This phenomenon co-occurs with the tropical cyclone season, resulting in strong rainfall events that can lead to catastrophic flooding in central Vietnam (15), the most recent occurring in October 2020 (16). While the summer and winter monsoons across Asia have been researched extensively (17–22), the limited instrumental records, climate model analyses, and paleoclimate records of autumn hydroclimate leave this important component of the Southeast Asian monsoon severely understudied (11, 12, 14).

Here, we present an autumn monsoon rainfall record from MSEA spanning 45 to 4 ka. We use a multiproxy stalagmite record from central Vietnam to show that the autumn monsoon underwent significant change over the last 45,000 y. Paired with climate model simulations, we find that glacially modulated sea level, a previously unknown control on MSEA hydroclimate, is a dominant driver of autumn monsoon rainfall variability on millennial-orbital timescales.

## Results and Discussion

### Autumn Monsoon Rainfall Variability Inferred from a Central Vietnam Stalagmite.

**Fig. 1.**
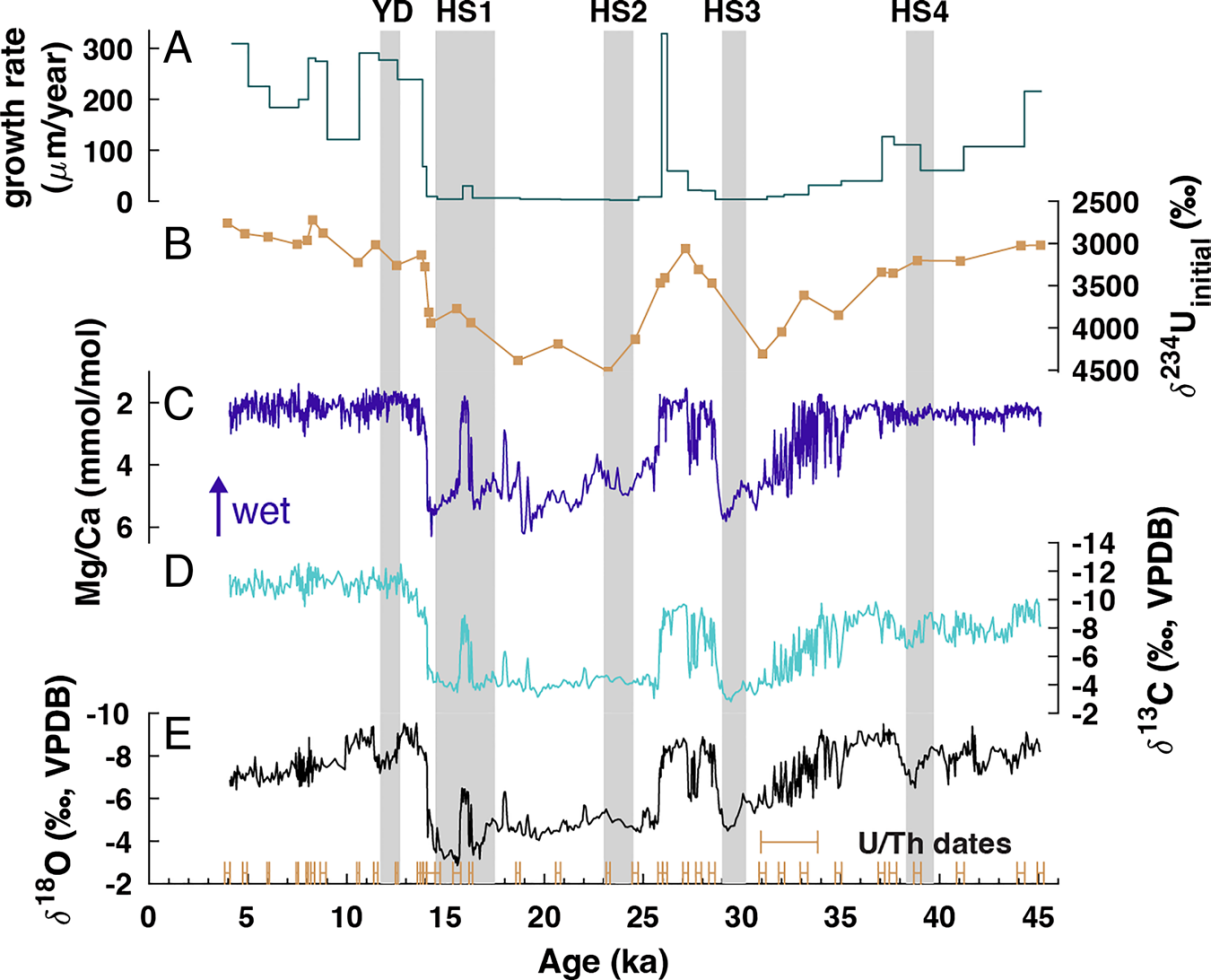
Time series of HH-1 proxies. Speleothem (*A*) growth rate (green), (*B*) δ^234^U_initial_ (tan), (*C*) Mg/Ca (blue), (*D*) δ^13^C (turquoise), and (*E*) δ^18^O (black) and 2σ error bars of U/Th ages (tan) from 4 to 45 ka. Gray shading denotes Heinrich Stadials 1 to 4 (HS) and the Younger Dryas (YD).

For this study, we use a previously broken 3.7 m-long stalagmite, HH-1 (*SI Appendix*, Fig. S3*A*), collected from Hoa Huong cave, a hydrologically active cave in Phong Nha-Ke Bang National Park in central Vietnam (17.5°N, 106.2°E; Elev. 411 m; See *SI Appendix*). Unlike the majority of MSEA, which primarily receives rainfall during the summer (JJA), from 1951 to 2007, our study site in central Vietnam received nearly half of its annual rainfall (~47%) during the autumn monsoon (SON) and only ~30% during JJA (*SI Appendix*, Fig. S1 *C* and *D*) (23). The HH-1 age model was constrained in time with 35 U–Th dates (*Methods* and *SI Appendix*, Fig. S3*C* and Table S1) and shows that it grew continuously between 45.1 and 4.0 ka, but at a highly variable growth rate (Fig. 1*A*). The fastest rates occur after the deglaciation (120 to 329 µm/y), while the slowest rates occur during HS 1 to 3 and the Last Glacial Maximum (LGM, ~20 ka; 2 to 10 µm/y; Fig. 1*A*). The HH-1 record consists of 768 carbon (δ^13^C) and oxygen (δ^18^O) stable isotope analyses and 1,384 Mg/Ca analyses, resulting in an average resolution of ~50 y and ~30 y, respectively (Fig. 1 *C*–*E* and *Methods*).

Similar to many studies (2, 24, 25), we interpret speleothem δ^13^C and Mg/Ca as tracers of local hydroclimate. The remarkable synchronicity of HH-1 δ^13^C and Mg/Ca records suggests a common control (Fig. 1), which we propose to be prior calcite precipitation (PCP). PCP is caused by CO_2_ degassing in the epikarst and within the cave and affects both speleothem δ^13^C and Mg/Ca (26). During PCP, lighter isotopes (^12^C) and calcium are preferentially removed from solution, leaving the remaining dripwater, and in turn speleothem carbonate, enriched in heavier isotopes (^13^C) and trace elements (Mg). Generally, periods of high PCP (high δ^13^C and Mg/Ca) reflect dry conditions and periods of low PCP (low δ^13^C and Mg/Ca) reflect wet conditions (2). In addition to PCP, there are several other known controls on speleothem δ^13^C such as vegetation type, soil respiration rate, atmospheric CO_2_ concentration, and open vs. closed system dissolution (27–30), which are difficult to deconvolve without multiproxy analyses. We point to the covariation between HH-1 δ^13^C and Mg/Ca (r = 0.89, *P* < 0.01), a robust indicator of PCP (2, 24, 25), as further evidence that PCP is likely the dominant control on both proxies. HH-1 Sr/Ca, a proxy also affected by PCP (25), also shows similarities with the Mg/Ca record (*SI Appendix*, Fig. S4). However, the records are antiphased, opposite to the expected in-phase relationship between Mg/Ca and Sr/Ca (26), suggesting that PCP does not drive HH-1 Sr/Ca. Due to the similarities between HH-1 Sr/Ca and growth rate (*SI Appendix*, Fig. S4), another known control on speleothem Sr/Ca (31), we propose that growth rate dominates HH-1 Sr/Ca. Because it is challenging to interpret climate from growth rate changes of a single stalagmite, we choose to focus our analyses on the PCP-driven Mg/Ca and δ^13^C records.

We use speleothem δ^234^U_initial_ values from our 35 U-series measurements to provide an independent measure of hydroclimate that is not affected by PCP but rather water–rock interaction and weathering (25, 32). ^234^U, produced by alpha decay of ^238^U via two short-lived intermediate daughter isotopes, may be directly ejected into solution during decay or more easily leached from radiation-damaged crystal lattice sites (i.e., alpha recoil), causing most natural water samples to deviate from secular equilibrium and exhibit a (^234^U/^238^U) activity ratio greater than 1 (33–35). During dry periods, slower infiltration rates may further elevate (^234^U/^238^U), increasing δ^234^U_initial_ in infiltrating water due to increased water–rock interaction times and/or accumulation and subsequent flushing of ^234^U in the epikarst (25, 36, 37). During wet periods, increased bedrock dissolution and/or faster infiltration may lead to lower δ^234^U_initial_. The covariation between the δ^234^U_initial_ and the δ^13^C and Mg/Ca records serves as additional evidence for our use of δ^13^C and Mg/Ca as hydroclimate proxies (Fig. 1). The similarities between these three proxies (δ^13^C, Mg/Ca, and δ^234^U_initial_) indicates that hydroclimate, rather than in cave processes, controls PCP and thus the δ^13^C and Mg/Ca signals.

As with other speleothem records from low-latitude regions, we expect HH-1 δ^18^O to primarily reflect variations in precipitation δ^18^O (δ^18^O_p_). For most of MSEA, the main control on interannual to orbital-scale δ^18^O_p_ variability is upstream rainout during the summer monsoon, an indicator of large-scale summer monsoon intensity (38, 39). Despite the different rainfall seasonality in central Vietnam, the average δ^18^O_p_ seasonal cycle is very similar to the majority of MSEA, with more negative values occurring during the summer months and more positive values occurring during the winter months (*SI Appendix*, Fig. S1*H*). However, because central Vietnam receives the majority of its rainfall during autumn, the controls on weighted mean annual δ^18^O_p,_ and hence cave dripwater δ^18^O, are more complex and likely reflect the relative contribution of summer (more negative δ^18^O) vs. autumn (more positive δ^18^O) rainfall. This signal is closely linked to the seasonal migration of the Intertropical Convergence Zone (ITCZ), which changes the annual contribution of isotopically light summer precipitation sourced from the Bay of Bengal and Indian Ocean vs. isotopically heavier autumn precipitation sourced from the South China Sea and the Western Pacific  (11). Finally, it is possible that tropical cyclones may also influence δ^18^O_p_ on short timescales, though we expect that mean annual δ^18^O_p_ in central Vietnam is likely dominated by large-scale changes to atmospheric circulation rather than local rainfall events. Nevertheless, we find that HH-1 δ^18^O shows a strong relationship with both δ^13^C and Mg/Ca (r = 0.79, 0.87, *P* < 0.01, respectively), suggesting that HH-1 δ^18^O may also reflect local hydroclimate change. If seasonality-driven changes in mean δ^18^O_p_ were the primary control, we might expect periods of increased autumn rainfall, as indicated by δ^13^C and Mg/Ca, to be associated with a more positive δ^18^O signal due to increased contribution of higher δ^18^O autumn rainfall, but we see the opposite relationship (locally wet periods exhibit more negative δ^18^O). An alternative explanation for the observed δ^18^O pattern is that PCP, another potential control on speleothem δ^18^O that has recently been proposed (40), may also influence HH-1 δ^18^O and explain the striking similarity between δ^18^O, δ^13^C, and Mg/Ca records. Because of these complex controls on δ^18^O_p_ and the potential for local PCP-driven changes to impact HH-1 δ^18^O, we focus our climatic interpretation on the Mg/Ca and δ^13^C records.

Using the δ^13^C and Mg/Ca records as tracers of local rainfall amount, we find that the Southeast Asian autumn monsoon underwent substantial change over the last 45,000 y (Fig. 1). Low Mg/Ca, δ^13^C, and δ^234^U_initial_ and high growth rate indicate relatively wet conditions prior to ~35 ka, followed by a gradual drying marked by increasing Mg/Ca, δ^13^C, and δ^234^U_initial_ and decreasing growth rates. There is a rapid dry–wet transition at the end of HS3 (~30 ka), which is followed by an abrupt return to dry conditions at ~25 ka. With the exception of a 500 y wet excursion at ~16 ka, dry conditions persist throughout the LGM and HS1. At ~14 ka, there is an abrupt dry–wet shift marking the transition to persistent wet conditions for the remainder of the record.

With an average temporal resolution of ~30 to 50 y, the HH-1 record best resolves centennial-orbital scale hydroclimate variability. Thus, the HH-1 record is not likely to reflect short-term El Niño Southern Oscillation (ENSO) variability, a known control of Southeast Asian hydroclimate on interannual-multidecadal timescales (5, 14, 41). On longer timescales, precessionally driven Indo-Pacific Warm Pool ocean heat content has been linked to ENSO as well as ocean–continent moisture transport in the Asian monsoon region (42, 43). However, the abrupt nature of change in the HH-1 record points toward different controls on central Vietnam hydroclimate, rather than the more gradual changes in ocean heat content.

**Fig. 2.**
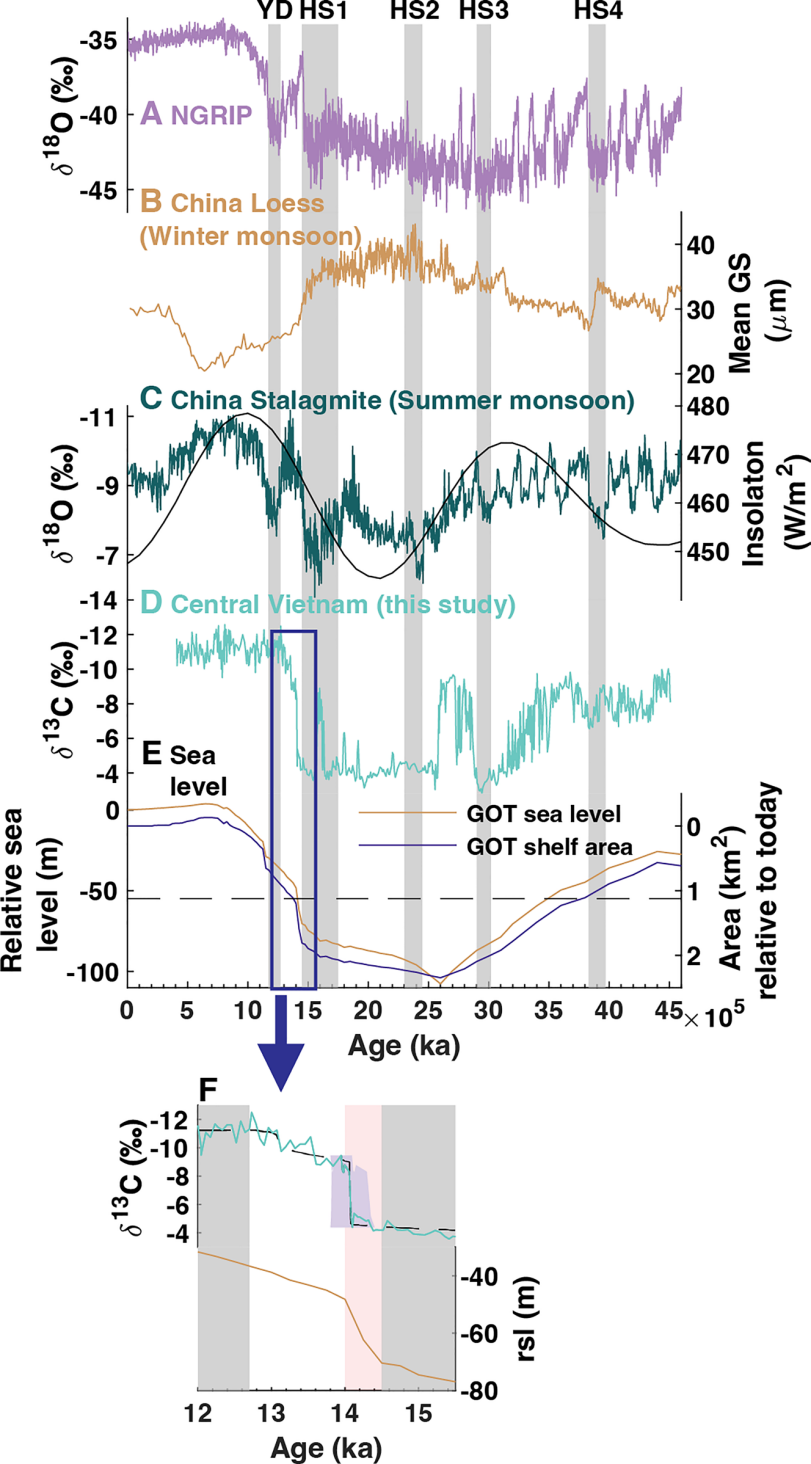
Comparison of HH-1 to regional records. (*A*) Ice core δ^18^O (VSMOW) from the North Greenland Ice Core Project (NGRIP; purple), (*B*) mean grain size (GS) of the Jingyuan loess sequence from the northwestern Loess Plateau in China (tan) (45), (*C*) composite stalagmite δ^18^O (VPDB) record from China (green) (17), (*D*) HH-1 δ^13^C (VPDB; turquoise), and (*E*) Gulf of Tonkin (GOT) relative sea level (yellow) and shelf area (blue) reconstructed from the ICE-PC2 data product (46). GOT shelf threshold of 112,000 km^2^ plotted as black dashed line. (*F*) Highlights a period of rapid change in HH-1 δ^13^C (*Top*) coinciding with rapid relative sea-level rise (*Bottom*) during MWP1a (pink shading; 14 to 14.5 kyrs BP). Black dashed curve in the top panel shows changepoints calculated using a Bayesian change-point algorithm (47), which identifies a significant regime shift at 14.2 ka. The 2σ age model uncertainty (blue shading) shows that the δ^13^C shift occurs during or after MWP1a.

The appearance of millennial-scale events in the HH-1 record, such as HS (dry conditions) and some DO events (wet conditions) (Fig. 2), demonstrates that the Southeast Asian autumn monsoon is sensitive to changes in Atlantic Meridional Overturning Circulation. On orbital timescales, the HH-1 record shows little resemblance to East Asian summer or winter monsoon variability. Since precessionally driven changes in insolation drive both systems (20), our results indicate that insolation forcing does not exert a direct control on Southeast Asian autumn monsoon rainfall variability. Instead, HH-1 δ^13^C and Mg/Ca mirror changes in local sea level driven by global ice sheet volume changes (Fig. 2). Periods of higher sea level correspond to wetter conditions and vice versa. This manifests as wet conditions during the sea-level high stand of the late Marine Isotope Stage (MIS) 3 transitioning to a drying trend from 35 to 30 ka, which is followed by pronounced dry conditions during the LGM sea-level low stand. An abrupt shift during Meltwater Pulse 1A (MWP1a; ~14.5 to 14 ka), a period of rapid sealevel rise, punctuates the dry period, which is followed by wet conditions and higher sea level for the remainder of the record. While sea-level change is a known driver of Indo-Pacific hydroclimate (8–10, 44), few studies explore this relationship in MSEA (6, 7). Notably, most studies focus on hydroclimate changes associated with the inundation of the Sunda and Sahul shelves during the deglaciation, which largely flooded after MWP1a, with the most rapid change occurring after the Younger Dryas (YD; 12.9 to 11.7 ka) (*SI Appendix*, Fig. S5*A*). An important difference in the HH-1 Mg/Ca and δ^13^C records is that they show no hydroclimate response during or after the YD, but instead exhibit a large shift at ~14.2 ka, ~ 2 ka earlier than the YD. In fact, the HH-1 record better resembles the inundation history of the Gulf of Tonkin and the South China Shelf, located northeast of the study site, which first flood during MWP1a (Fig. 2*E* and *SI Appendix*, Fig. S5*A*). This indicates that local sea-level change may drive changes in Southeast Asian autumn monsoon rainfall rather than the more distally located Sunda and Sahul shelves. Additionally, the Sunda/Sahul region is not a moisture source of modern central Vietnam rainfall (*SI Appendix*, Fig. S2), suggesting that the exposure/inundation of these landmasses is unlikely to affect moisture delivery to central Vietnam.

### Deglacial Autumn Monsoon Rainfall Variability Characterized by Rapid Centennial-Scale Changes.

Rapid mean-state change is a notable feature of the HH-1 record. Two prominent dry–wet shifts occur at the end of HS3 (~30 ka) and during MWP1a, a period associated with rapid sea-level rise (Fig. 2). To further investigate the mechanisms behind these shifts, we utilize iTRACE, an isotope-enabled transient climate model simulation which spans the deglaciation 20 to 11 ka (*Methods*). We focus our analyses on the transition between HS1 and the Bølling–Allerød (BA) interstadial because this period encompasses substantial change in both sea level and meltwater flux. Note, we use the term *meltwater forcing* to refer to freshwater fluxes into the North Atlantic during HS and the Younger Dryas. Meltwater forcing minimally contributes to sea-level change, which is instead primarily driven by large-scale changes in continental ice sheet volume (48).

**Fig. 3.**
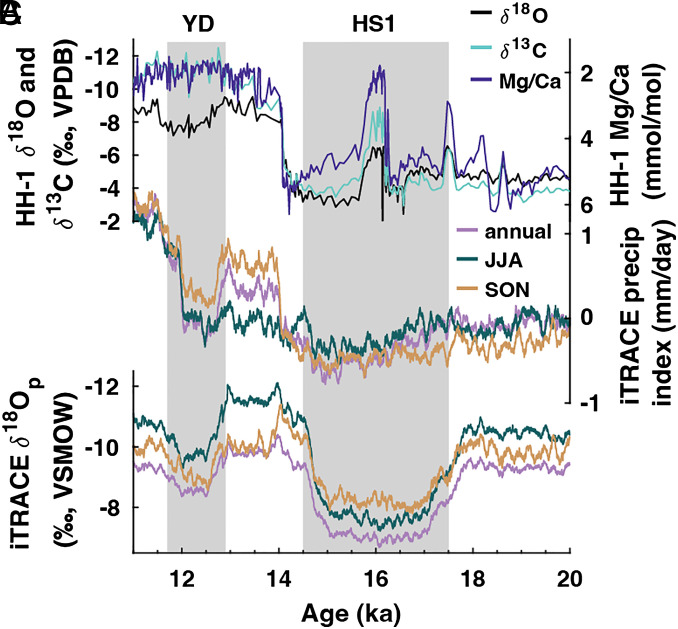
Comparison of HH-1 to iTRACE time series from 11 to 20 ka. (*A*) HH-1 geochemical proxies, (*B*) iTRACE precipitation (index), and (*C*) iTRACE weighted δ^18^O_p_. In *B* and *C*, we plot the annual mean (light purple), JJA (green), and SON (tan) from the grid cell encompassing the study site. We smoothed the iTRACE time series with a 100 y running mean for aesthetic purposes and readability.

**Fig. 4.**
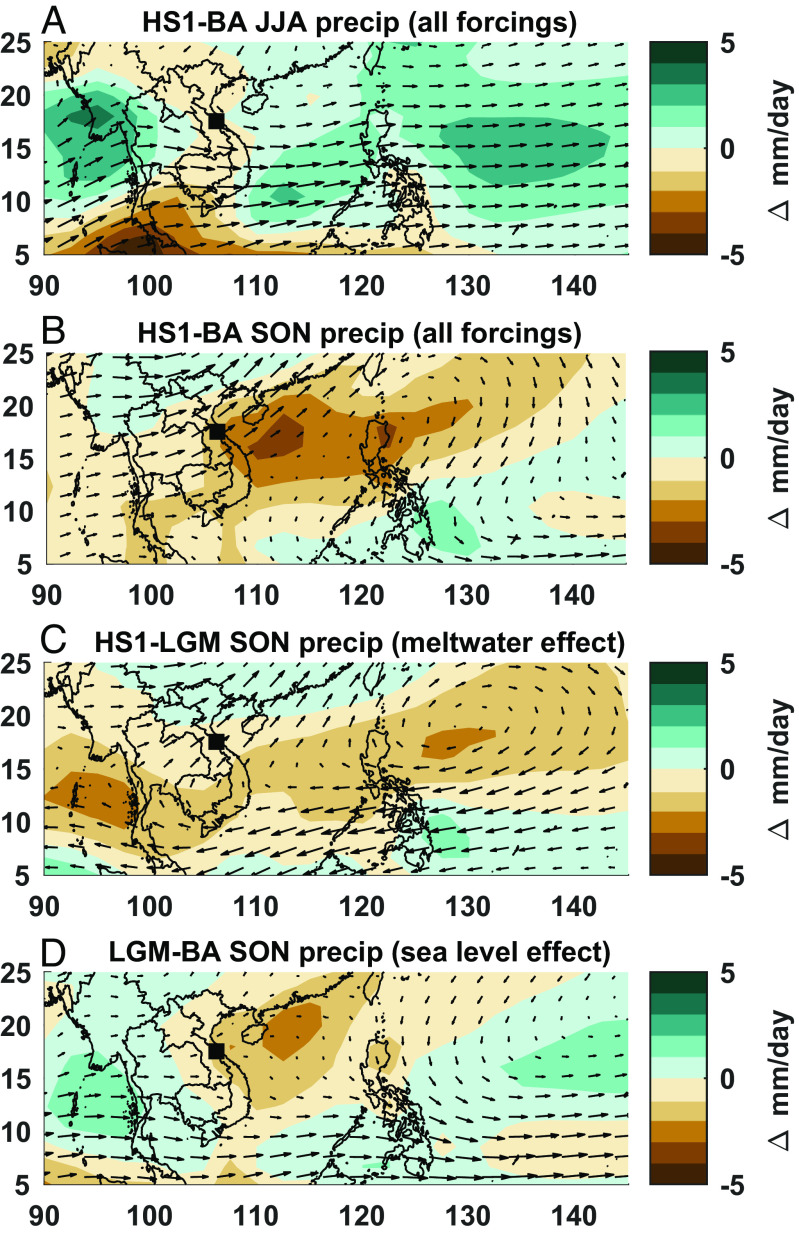
iTRACE precipitation and 850 mbar wind anomalies over MSEA. (*A*) and (*B*) show anomalies between HS1 and BA for JJA and SON, respectively. (*C*) HS1-LGM isolates the meltwater effect on SON precipitation during HS1 by subtracting the LGM, a baseline cold period with no meltwater forcing, from HS1. (*D*) LGM-BA isolates the sea-level effect on SON precipitation by subtracting the LGM, a period of low sea level and no meltwater forcing, from BA, a period of high sea level. The black square in all panels denotes the location of Hoa Huong cave.

Simulated precipitation from the grid cell encompassing Hoa Huong cave closely matches the HH-1 speleothem record (Fig. 3 *A* and *B*). Similarities include dry conditions throughout the LGM and HS1 and an abrupt shift to wet conditions at ~14 ka. The iTRACE simulations reveal that SON precipitation, rather than JJA precipitation, drives the jump at 14 ka observed in the speleothem record (Fig. 3*B*). Indeed, the similarities between simulated precipitation in iTRACE and the HH-1 record support our interpretation of HH-1 being a robust proxy for autumn monsoon rainfall and demonstrate the strong control of SON precipitation variability on MSEA hydroclimate. On a regional scale, this shift (BA-HS1) manifests as a pronounced dry pattern during SON centered over the Northern South China Sea and extending into eastern MSEA (Fig. 4*B*), whereas JJA precipitation change only causes weak drying in eastern MSEA (Fig. 4*A*).

Using the iTRACE simulations, we attribute the SON drying pattern to two different mechanisms related to meltwater forcing and sea-level change (Fig. 4 *C* and *D*). To isolate the effect of meltwater forcing, we assess the difference between the LGM (i.e., no meltwater forcing) and HS1 (i.e., enhanced meltwater forcing). The resulting pattern reveals anomalously dry conditions during HS1 across the Northern South China Sea and Western Pacific, which is caused by an anomalous anticyclone that advects dry air masses over eastern MSEA (Fig. 4*C*). He et al. (13), first described this phenomenon where a weakening of the Atlantic Meridional Overturning Circulation causes the convergence of anomalous northerly and southerly winds over South China during SON, creating a north–south precipitation dipole across South China (wet) and MSEA (dry). The antiphasing of the HH-1 record and the South China speleothem records is consistent with this dipole pattern (*SI Appendix*, Fig. S6). We find that this relationship even extends to the 16 ka wet excursion in the HH-1 record (dry in South China), a feature unexplained by the iTRACE simulations, demonstrating some regional coherence for this event. The dry conditions during HS2 (Mg/Ca), HS3 (δ^13^C and Mg/Ca), and HS4 (δ^13^C) indicate that this mechanism persisted beyond the last deglaciation (Fig. 2). However, we find that the Mg/Ca and δ^13^C proxies do not consistently record dry conditions during all Heinrich Stadials, suggesting that drying from this mechanism may be limited. For example, we attribute the absence of drying during HS2 in the δ^13^C record to the co-occurrence of the LGM, an already dry period in the record, which may obscure the drying effects from meltwater forcing. Similarly, neither Mg/Ca nor δ^13^C showed particularly dry conditions during HS1 when compared to the LGM, suggesting that both proxies may be less sensitive once conditions are already dry.

The 14 ka autumn precipitation jump in the iTRACE simulations corresponds to a change in global sea level (ICE curve in *SI Appendix*, Fig. S7*B*) when the Gulf of Tonkin and the South China Shelf were flooded (*SI Appendix*, Fig. S8). The abrupt shift in the HH-1 record (14.2 ka) occurs slightly before this shift in the model (14 ka), but still falls within the timing of MWP1a, which is the most rapid period of global and local sea-level rise during the deglaciation (Fig. 2*F*). When we isolate the effect of this sea-level forcing (BA-LGM), the iTRACE simulations reveal a strong regional drying pattern encompassing much of the Northern South China Sea, eastern MSEA, and South China (Fig. 4*D*). The strongest drying occurs over the Gulf of Tonkin and a shelf in South China, exposed landmasses during the LGM. iTRACE simulates anomalous subsidence over this region (ω > 0) (*SI Appendix*, Fig. S9). Similar to the drying mechanism over the Maritime Continent during the LGM (9, 49), this exposed land cools more than the adjacent ocean, reducing convection (*SI Appendix*, Fig. S9). This decrease in moisture ultimately reduces MSEA SON rainfall, as this region lies northeast of MSEA, along the moisture transport pathway of the Southeast Asian autumn monsoon (*SI Appendix*, Fig. S2*C*).

To summarize, we attribute the abrupt dry–wet shift at the end of HS3 to meltwater forcing, whereas the shift at 14.2 ka is primarily driven by rapid sea-level rise during MWP1a. However, it is worth noting that neither the sea level nor meltwater mechanisms explain the abrupt drying at 25 ka or the wet excursion during HS1 (~16 ka). We propose that the wet conditions during 30 to 25 ka reflect the two DO events that occur during this time, which may have created wet conditions before returning to dry conditions at ~25 ka. The 16 ka event likely reflects a more localized hydroclimate shift, with some evidence that it is a regional event spanning from central Vietnam to South China (*SI Appendix*, Fig. S6). Another notable discrepancy is the shift to dry conditions in the iTRACE simulations during the YD, a feature that does not appear in the δ^13^C or Mg/Ca records. Similar to BA-HS1 drying, iTRACE analyses reveal that both meltwater forcing and sea-level rise contribute to the dry–wet transition at the end of the YD (*SI Appendix*, Fig. S10). This discrepancy indicates that iTRACE may overestimate drying during this period, or that the δ^13^C or Mg/Ca proxies at our cave site are not sensitive enough to detect this change. Notably, HH-1 δ^18^O does increase during the YD. Since the controls on δ^18^O are unclear, this feature could reflect local drying and/or large-scale changes to atmospheric circulation.

Consistent with previous findings that local rainfall amount does not control central Vietnam δ^18^O_p_ (11), weighted iTRACE δ^18^O_p_ and precipitation show some important differences. Unlike iTRACE precipitation and the HH-1 local hydroclimate proxies (Fig. 3 *A* and *B*), iTRACE δ^18^O_p_ does not exhibit an abrupt shift associated with sea-level rise during MWP1a (Fig. 3*C*). Instead, changes in iTRACE δ^18^O_p_ show ties to meltwater forcing, with values increasing at the onset and decreasing at the end of HS1. On a spatial scale, iTRACE δ^18^O_p_ does not resemble rainfall anomalies across MSEA (Fig. 4 *A* and *B* and *SI Appendix*, Fig. S11), further demonstrating the dissociation of local rainfall and δ^18^O_p_.

Since central Vietnam receives rainfall during summer and autumn, it is possible that summer rainfall contributes to changes in the HH-1 record. In addition to decreased autumn rainfall, decreased summer rainfall may add to the dry conditions during HS. iTRACE simulations reveal a weak meltwater-driven drying pattern over the study site during HS1 (Fig. 4*A*, MWF curve in *SI Appendix*, Fig. S7*A*). This is a well-known phenomenon, where weakened Atlantic Meridional Overturning Circulation during HS shifts the ITCZ and the westerly jet south, weakening the Asian summer monsoon and changing rainfall patterns across the region (50, 51). Conversely, we do not expect sea-level change at the Gulf of Tonkin and South China Shelf to affect summer precipitation because the moisture source and transport pathway lie west of the study site (*SI Appendix*, Fig. S2*B*). While the inundation/emergence of the Sunda Shelf does affect summer rainfall across MSEA (6), we reiterate that the Sunda Shelf inundation history does not match the hydroclimate change documented in the HH-1 record (*SI Appendix*, Fig. S5*A*). For this reason, we suggest that hydroclimate changes due to sea-level change reflect shifts in autumn monsoon rainfall.

### Sea-Level Change Modulates MSEA Autumn Monsoon Rainfall Variability.

On orbital timescales, local sea-level change is the dominant driver of Southeast Asian autumn monsoon rainfall variability. HH-1 does not linearly track relative sea-level change, but rather follows the inundation history of the Gulf of Tonkin and the South China Shelf (Fig. 2*E* and *SI Appendix*, Fig. S8 *D* and *E*). Wet conditions from 45 to 35 ka and 13 to 4 ka correspond to periods when these landmasses were largely inundated. The driest conditions occur during the LGM, when sea level is lowest (Fig. 2*E*). The drying trend during late MIS 3 from 35 to 30 ka occurs during a period when global mean sea level dropped >40 m (*SI Appendix*, Fig. S5*B*) (52), exposing the Gulf of Tonkin and the South China Shelf (*SI Appendix*, Fig. S8*E*). The abrupt shift to wet conditions during MWP1a occurs during rapid regional inundation from 14.5 to 14 ka (*SI Appendix*, Fig. S8*D*). Wetting conditions continue in the HH-1 record from 14 to 13 ka, but at a slower rate than during MWP1a, matching a similar shift in the rate of local sea-level rise (Fig. 2*F*). Notably, sea level is higher prior to 35 ka and after 13 ka, but we do not see a corresponding wetting trend in the HH-1 proxies. Instead, the HH-1 Mg/Ca and δ^13^C records appear to reach a lower limit, indicating that there may be a threshold where shelf exposure no longer contributes to regional drying. We note that this threshold occurs at a similar stage in sea-level history regardless of increasing or decreasing sea level, when the Gulf of Tonkin shelf exposure is ~112,000 km^2^ (dashed black line in Fig. 2*E*). Alternatively, this limit may reflect a threshold in the proxies themselves, where they are no longer sensitive to wetter conditions (i.e., PCP = 0).

While sea level is not the only driver of Southeast Asian autumn monsoon rainfall variability, its relative state (high or low) appears to modulate centennial- and millennial-scale events in the HH-1 record. This is most obvious during wet events, which are dampened during sea-level highstands and amplified during low sea level. For example, the DO events during high sea level (45 to 35 ka) result in ~1‰ negative excursions in δ^13^C record, whereas the two DO events during low sea level coincide with a ~6‰ negative excursion from 30 to 25 ka (Fig. 2). Other anomalies in the HH-1 record, including the wet excursion during HS1 and pronounced variability during the 35 to 30 ka drying trend, further demonstrate how a lower sea level contributes to a more volatile hydroclimate in central Vietnam. The relationship between sea level and dry events is less clear. However, the strongest drying response to meltwater forcing in the δ^13^C record occurs during HS4, a period of high sea level. The drying during Heinrich Stadials 1, 2, and 3 is more muted, suggesting that the stronger sea-level mechanism may obscure the weaker meltwater drying signal associated with Heinrich Stadials.

In conclusion, our multiproxy record reveals that sea level is the main driver of Southeast Asian autumn monsoon rainfall variability on orbital timescales. Changes in sea level and meltwater forcing lead to abrupt hydroclimate change in central Vietnam, demonstrating the important role autumn rainfall plays in MSEA across a variety of timescales. Finally, the agreement between climate model analyses and the HH-1 record adds confidence that climate projections may correctly simulate the Southeast Asian autumn monsoon, improving our understanding of how rainfall changes might affect this densely populated region.

## Methods

### U–Th Dating and Age Model Construction.

We drilled 36 samples for U–Th dates from the central growth axis of stalagmite HH-1 using a handheld rotary tool with a diamond bur. U–Th measurements were conducted at Massachusetts Institute of Technology where powdered samples (~100 to 250 mg) were dissolved, spiked with a ^229^Th-^233^U-^236^U tracer, separated of U and Th by iron coprecipitation and elution in columns with AG1-X8 resin, and analyzed using a Nu Plasma II-ES multicollector-inductively coupled plasma-mass spectrometer equipped with an Aridus 2 desolvating nebulizer, following methods described in the study by Burns et al. (53). We corrected raw ages for initial ^230^Th using an initial ^230^Th/^232^Th value of 25 ± 12.5 ppm. All dates were in stratigraphic order (*SI Appendix*, Table S1), except for UU-H12, which we excluded from the final age model. The top and bottom dates were 3,961 and 45,109 yrs B.P., respectively. To construct the HH-1 age model and 95% CI (*SI Appendix*, Fig. S3*C*), we performed 2,000 Monte-Carlo simulations via the COPRA age-depth modeling software (54).

### Geochemical Analyses.

We drilled samples for geochemical analyses along the central axis of HH-1. Since the growth rate of HH-1 ranges from 2 to 329 µm/yr, we opted to vary the sampling interval dependent on growth rate. For fast-growing portions of the stalagmite, we sampled at 2.5 to 5 mm intervals using a handheld rotary tool fitted with a 1-mm diameter diamond bur. We sampled slow-growing portions of the stalagmite continuously at 0.25 to 0.5 mm resolution using a Sherline micromill.

We analyzed 1,384 samples for Mg/Ca on an Nu Instruments Attom High-Resolution-Inductively Coupled Plasma-Mass Spectrometer at the Center for Isotope Tracers in Earth Science laboratory at the University of California, Irvine [average uncertainty (1σ) = 3%]. Raw intensities were converted to concentrations utilizing an external calibration curve constructed using five standards of known concentration and a blank. To correct for instrument drift, we spiked all samples, standards, and blanks with an internal Ge standard prior to analysis. The final HH-1 Ma/Ca record has an average resolution of ~30 y.

Due to the similarities between the Mg/Ca and isotope records, we chose to analyze a subset of 768 samples for stable isotopes, which yields an average resolution of ~50 y. Stable carbon and oxygen isotope measurements were performed at the University of Arizona Stable Isotope Lab, using a KIEL-III Carbonate Device coupled to a Finnigan MAT252 isotope ratio mass spectrometer (1σ of ± 0.11‰ for δ^18^O, 0.08‰ for δ ^13^C) and in the Department of Earth and Planetary Sciences, Rutgers University, using a Multiprep device attached to a MicroMass Optima Mass Spectrometer. Isotope ratios were calibrated to repeated measurements of NBS-19 and NBS-18 at the University of Arizona and to an in-house laboratory reference material (RGF1) at Rutgers, which is routinely calibrated to NBS19 at Rutgers. We applied a small correction in the University of Arizona δ^18^O measurements, which are offset by +0.2‰ relative to the Rutgers measurements. Values were reported in VPDB.

### iTRACE.

iTRACE is a water isotope–enabled transient simulation of the last deglaciation (11 to 20 ka) (50). iTRACE simulations are performed in iCESM1.3 (55), which is comprised of Community Atmosphere Model version 1.3 (CAM5.3), the Community Land Model version 4 (CLM4), Parallel Ocean Program version 2 (POP2), and Los Alamos Sea Ice Model version 4 (CICE4). The resolution of the land and atmosphere is 1.9° × 2.5° (latitude and longitude) with 30 vertical levels in the atmosphere. iCESM 1.3 simulations are well validated against modern observations of global precipitation and precipitation isotopes (55–57). When compared to modern observations, preindustrial iCESM 1.3 simulations closely capture seasonal changes in rainfall and *δ*^18^O_p_ across MSEA and central Vietnam, including the transition from the SW summer monsoon to the NE autumn monsoon (*SI Appendix*, Fig. S1).

iTRACE uses four forcing factors (ICE: continental ice sheets from the ICE-6G model (58) and KMT ocean bathymetry, ORB: orbital forcing, GHG: greenhouse gas concentrations from ice core reconstructions (59–61), and MWF: meltwater fluxes (hosing) following TRACE-21 ka (62), which are applied additively to create four parallel simulations (ICE, ICE + ORB, ICE + ORB + GHG, and ICE + ORB + GHG + MWF). The first three simulations start at 20 ka, whereas the MWF experiment begins at 19 ka. Ice sheet configuration changes in 1,000 y increments and ocean bathymetry are modified twice at 14 ka and 12 ka based on changes in global sea level from the ICE-6G model (*SI Appendix*, Fig. S8 *A*–*C*). At 14 ka, the Gulf of Tonkin and the shelf in South China are flooded. At 12 ka, the Sunda and Sahul shelves are flooded. To approximate the effect of a single forcing, we subtracted simulations from each other [e.g., ORB = (ICE + ORB)–ICE]. Refer to ref. 50 for additional details on the forcings. In this study, we compare the mean state of several time periods including 11.1 ka (11.1 to 11 ka, the last 100 y of the simulation), the YD (12.7 to 12.4 ka), the BA (13.8 to 13.3 ka), HS1 (15.5 to 15 ka), and the LGM (20 to 19 ka).

### Sea Level and Paleoshoreline Reconstruction.

To calculate relative sea-level change at the Gulf of Tonkin, we used a gravitationally self-consistent glacial isostatic adjustment model. Our calculations are based on the theory and pseudo-spectral algorithm described by Kendall et al. (63) with a spherical harmonic truncation at degree and order 512. These calculations include the impact of load-induced Earth rotation changes on sea level (64, 65), evolving shorelines, and the migration of grounded, marine-based ice (63, 66–68).

Our numerical predictions require models for Earth’s viscoelastic structure and the history of global ice cover. We adopted the viscosity profile VM2 (69) and the ICE-PC2 global ice history (46) to reconstruct relative sea level and paleoshorelines. The ICE-PC2 global ice history is characterized by a peak GMSL value during MIS 3 of −37.5 m at 44 ka, following Pico et al. (70), and −15 and −10 m during MIS 5a and 5c, respectively, which are within the ranges derived by Creveling et al. (71). We plot relative sea level from the nearest present-day ocean grid cell east of the study site (~18°N, 107°E) and calculated the inundation history of the Gulf of Tonkin within the area 15 to 23°N, 105 to 112°E (Fig. 2*E*). To reconstruct paleoshorelines, we subtracted the predicted relative sea-level change of a particular time step from the present-day topography (BOCD GEBCO dataset, https://www.gebco.net/). ICE-PC2 interpolates sea level between MIS 3 and the LGM. To address this uncertainty, we also compare the HH-1 record to a coral-based global mean sea-level reconstruction (*SI Appendix*, Fig. S5*B*) (52), a more detailed record of sea-level change during this time.

## Supplementary Material

Appendix 01 (PDF)

## Data Availability

Time series data have been deposited in the National Oceanic and Atmospheric Administration National Centers for Environmental Information Paleoclimatology archive (https://www.ncei.noaa.gov/access/paleo-search/study/38139) (72).
