# Supplementary material for: Glacial changes in sea level modulated millennial-scale variability of Southeast Asian autumn monsoon rainfall
Source: Proc Natl Acad Sci U S A. 2023 Jun 26;120(27):e2219489120. doi: 10.1073/pnas.2219489120 (PMC10319016; doi:10.1073/pnas.2219489120)
Supplement: Supplementary file 1 — Appendix 01 (PDF) [file pnas.2219489120.sapp.pdf]

PNAS

**Supporting Information for**

Glacial changes in sea level modulated millennia-scale variability of Southeast Asian autumn monsoon rainfall

Elizabeth W. Patterson[1], Kathleen R. Johnson[1], Michael L. Griffiths[2], Christopher W. Kinsley[3,4], David McGee[4], Xiaojing Du[5], Tamara Pico[6], Annabel Wolf[1], Vasile Ersek[7], Richard A. Mortlock[8], Kweku A. Yamoah[9], Thành N. Bùi[10], Mùi X. Trần[10], Quốc Đỗ-Trọng[11], Trí V. Võ[10], Trí H. Đinh[10]

[1]Department of Earth System Science, University of California, Irvine, CA 92617, USA
[2]Department of Environmental Science, William Paterson University, Wayne, NJ 07470, USA
[3]Berkeley Geochronology Center, Berkeley, CA 94709, USA
[4]Department of Earth, Atmospheric and Planetary Sciences, Massachusetts Institute of Technology, Cambridge, MA 02139, USA
[5]Department of Earth, Environmental, and Planetary Sciences, Brown University, Providence, RI 02912, USA
[6]Department Earth and Planetary Sciences, University of California, Santa Cruz, CA 95064, USA
[7]Department of Geography and Environmental Sciences, Northumbria University, Newcastle, NE1 8ST, UK
[8] Department Earth and Planetary Sciences, Rutgers University, Piscataway, NJ 08854, USA
[9]BioArc, Department of Archaeology, University of York, York YO10 5DD, UK
[10]Phong Nha Ke Bang National Park, Phong Nha, Botrach, Quangbinh, VN
[11]University of Science, Vietnam National University, Hanoi, VN

*Elizabeth W. Patterson.
**Email:** epatter2@uci.edu

**This PDF file includes:**

Supporting text
Figures S1 to S11
Tables S1
SI References

## Supporting Information Text

### Cave and speleothem sample.

Hoa Huong Cave (HH cave) is a 3,910 m-long cave located 411 m above sea level in Phong Nha-Ke Bang National Park in Quang Binh Province, Vietnam (17.5°N, 106.2°E). Members from the Vietnam Caves Expedition group extensively surveyed HH cave in 2012 and 2016. HH cave has one known entrance, which intersects the continuous cave passageway. The thickness of bedrock overlying the cave ranges from a few meters close to the entrance to >100 meters in the deeper sections of the cave. Dense, tropical, evergreen forest comprise the vegetation overlying and surrounding HH cave.

We have conducted cave monitoring at HH cave since March 2020. Over two years of logger data reveal that mean cave temperature is 20 °C with a seasonal cycle of 1.5-2 °C. While mean annual temperature in the nearest town, Phong Nha, is ~23 °C, we believe that HH cave temperature still reflects mean annual temperature because of its higher elevation (>200 m higher than Phong Nha) and location in densely vegetated forest. Cave relative humidity is over 95% throughout the year. These relatively constant temperature and relative humidity conditions are favorable for stalagmite formation. Two cave air $pCO_2$ measurements from March 2020 (820ppm) and August 2022 (3120pm) suggest that HH cave may have seasonal ventilation. Additional $pCO_2$ measurements are needed to confirm the presence/or and magnitude of the seasonal cycle.

We collected the previously broken stalagmite, HH-1 (Fig. S3a,b), ~150 m from the cave entrance. There is no active drip above the base of HH-1, so no dripwater monitoring is possible at this specific location in the cave. The top of the sample and the ceiling of the cave show remnants of a soda straw, which was likely connected to the top of the stalagmite before it fell. HH-1 is comprised of dense calcite. While speleothem morphology remains relatively constant throughout the sample, indicating a constant drip rate (1), fabric changes coincide with changes in growth rate. Darker banding corresponds with slower growing portions of the stalagmite, whereas white or clear banding corresponds with faster growing portions (Fig. S3b). To confirm that HH-1 grew continuously, we sampled U-Th dates on either side of fabric changes (Table S1).

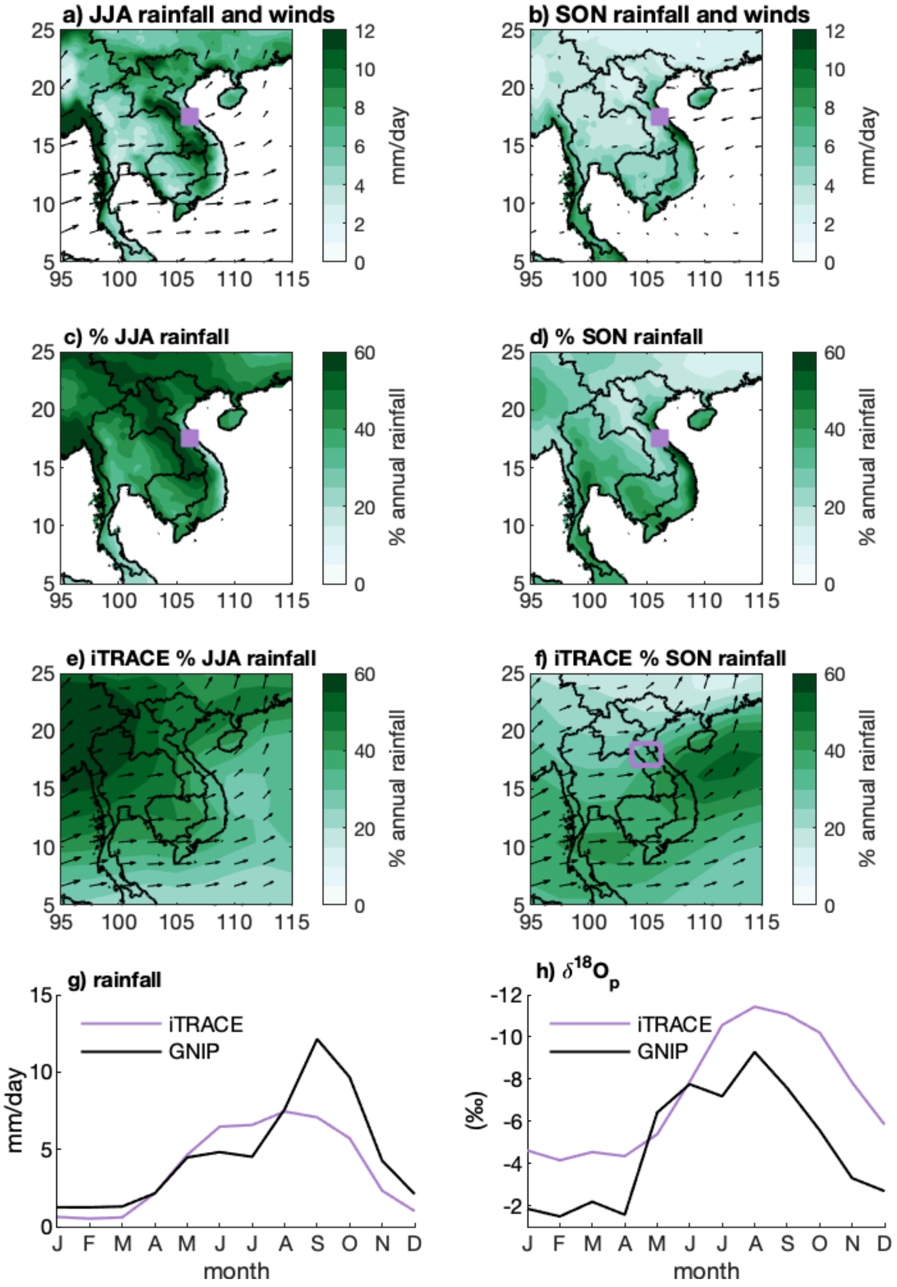


**Fig. S1.** Central Vietnam climatology and comparison to iTRACE. Average a) JJA and b) SON rainfall (1951-2007) derived from Asian Precipitation-Highly Resolved Observational Data Integration Towards Evaluation (APHRODITE)(2) and 850 mb winds (1951-2007) derived from NCEP reanalysis winds (3). Annual proportion of c) JJA and d) SON rainfall. The purple square denotes the location of Hoa Huong cave. Panels e) and f) are the same as c) and d) but show % annual rainfall and average 850 mb winds from the iTRACE preindustrial simulation. Seasonal cycle of g) rainfall from Aphrodite (black) and iTRACE (purple) and h) $\delta^{18}O_p$ from the GNIP station in Dong Hoi (black) and iTRACE (purple). iTRACE seasonal cycle derived from the grid cell encompasses HH cave (purple box in f).

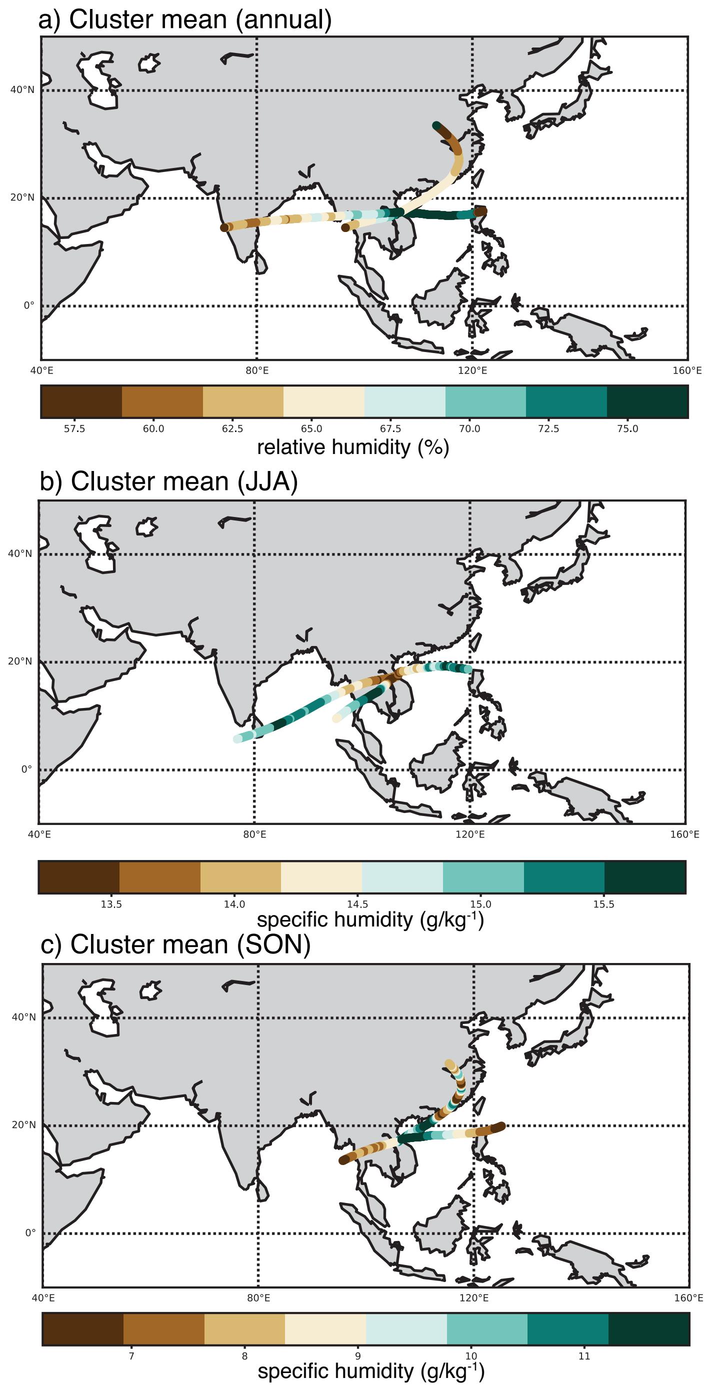


**Fig. S2.** Moisture source back-trajectory for rainfall in central Vietnam. Main Hybrid Single-Particle Lagrangian Integrated Trajectories (HYSPLIT) (4) cluster trajectories showing relative humidity for a) annual, and specific humidity for b) JJA, and c) SON. Clusters constructed from daily rain-bearing trajectories from 2006-2018 using meteoric parameters from the Global Data Assimilation System (GDAS) 0.5° dataset. We use the HYSPLIT model to calculate daily backward trajectories every 6 hours tracing for 120 hours, at a height of 1500 m above mean sea level. The simulated trajectories are used to calculate the cluster means using a cluster analysis method in HYSPLIT (5). Only rain-bearing trajectories are included in the cluster means. Evaporation and precipitation thresholds were set to -0.2 and 0.2, respectively.

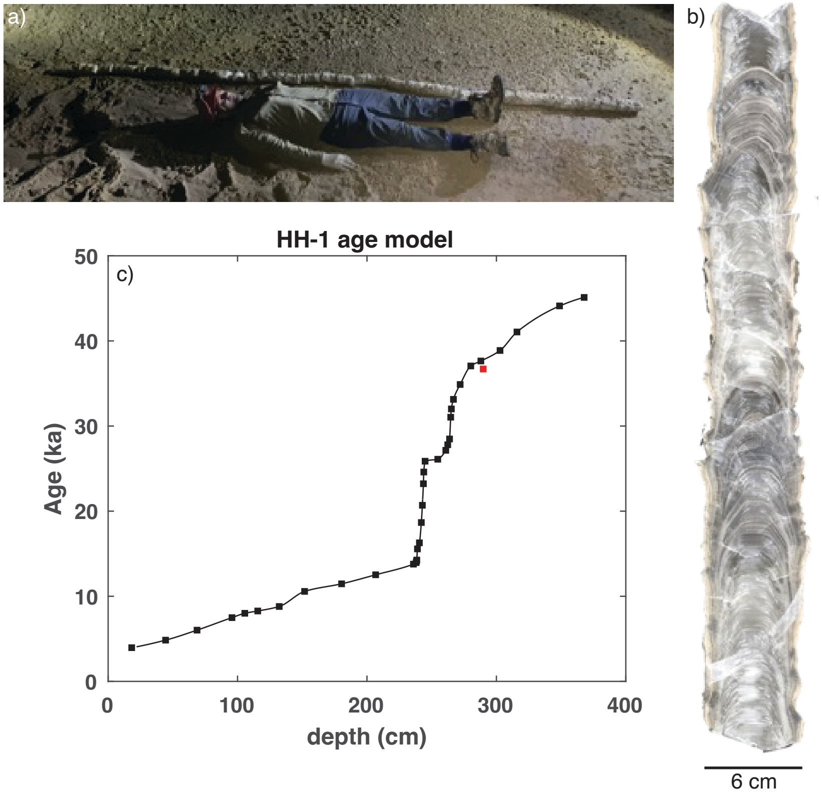


**Fig. S3.** HH-1 morphology and age model. a) Photo of HH-1 in HH cave. b) Scanned image of a portion of HH-1 to show representative fabric. c) HH-1 age-depth model. Black squares indicate U-Th dates and the red square indicates the one date excluded from the age model. The 2$\sigma$ error bars are plotted, but are smaller than the symbol. Age uncertainty can also be found in Table S1.

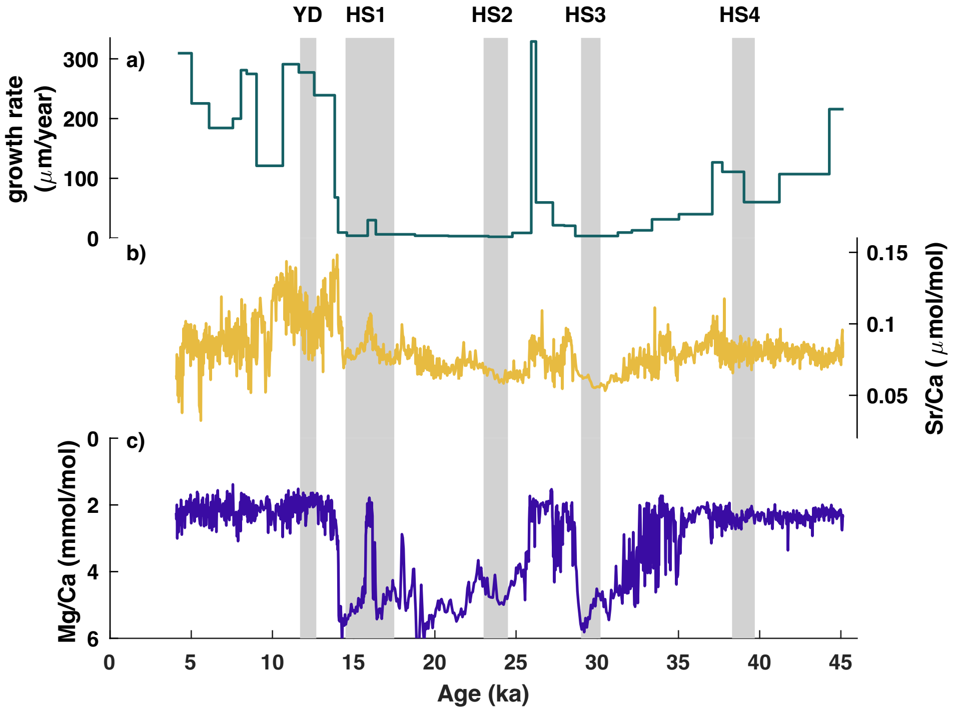


**Fig. S4.** HH-1 Sr/Ca vs other HH-1 proxies. a) growth rate (green), b) Sr/Ca (yellow), c) Mg/Ca (blue; note inverted axis).

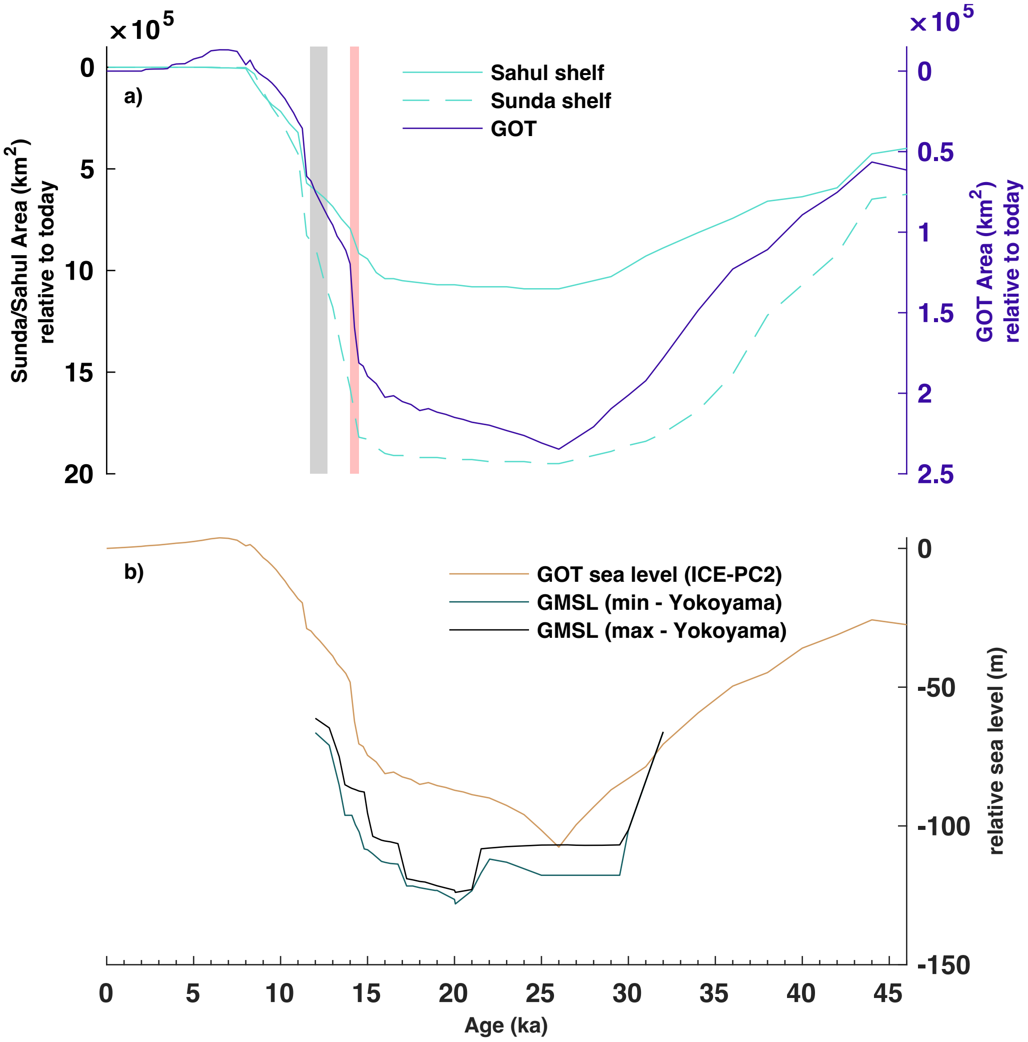


**Fig. S5.** Regional shelf exposure and sea level history. a) Shelf exposure curves of the Sahul Shelf (turquoise - solid), Sunda Shelf (turquoise - dashed), and the Gulf of Tonkin (GOT; dark blue) reconstructed from the ICE-PC2 data product (6). The pink shading denotes the timing of MWP1a (14 – 14.5 ka) and the grey denotes the YD (12.9 – 12.7 ka). The Sunda and Sahul shelf exposure curves are from (7). b) GOT relative sea level from ICE-PC2 data product (tan), and minimum (dark green) and maximum (black) estimates of global mean sea level (GMSL) from a coral-based reconstruction (8).

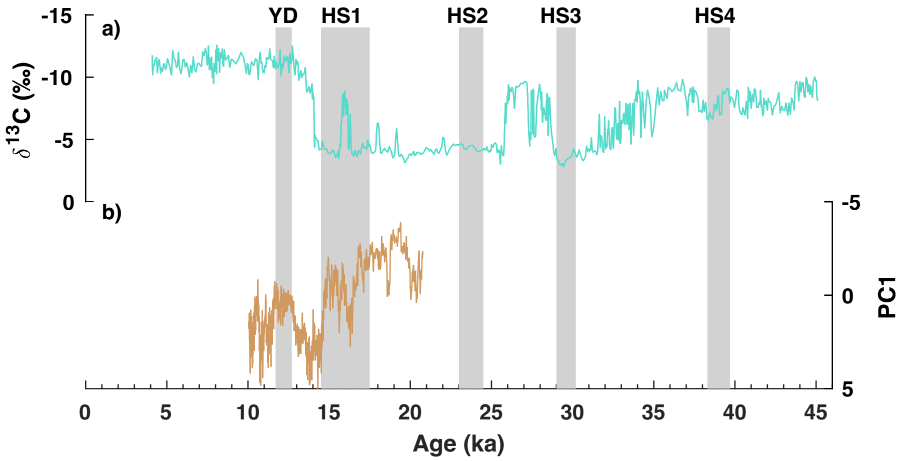


**Fig. S6.** Comparison of a) HH-1 $\delta^{13}C$ (turquoise**)** and b) PC1 of trace element records from two stalagmites from Haozhu Cave in central China (tan) (9).

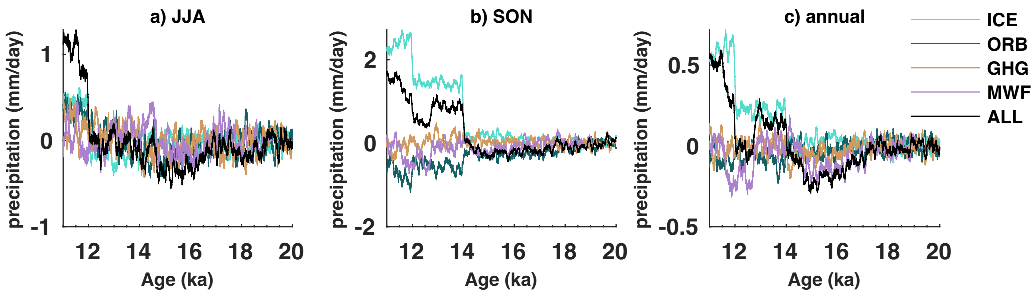


**Fig. S7.** Time series of individual forcings of simulated iTRACE precipitation. iTRACE a) JJA, b) SON, and c) annual precipitation time series from the grid cell encompassing the study site in response to different forcings (ICE - turquoise, ORB - green, GHG - tan, MWF - purple, and ALL forcings - black). We standardize the curves by subtracting the LGM climatology. The different forcings are isolated from the different iTRACE runs as follows; ICE = ICE – LGM, ORB = ICE + ORB – ICE – LGM , GHG = ICE + ORB + GHG – ICE + ORB – LGM, and MWF = ICE + ORB + GHG + MWF – ICE + ORB + GHG – LGM. Curves are smoothed with a 100 year running mean.

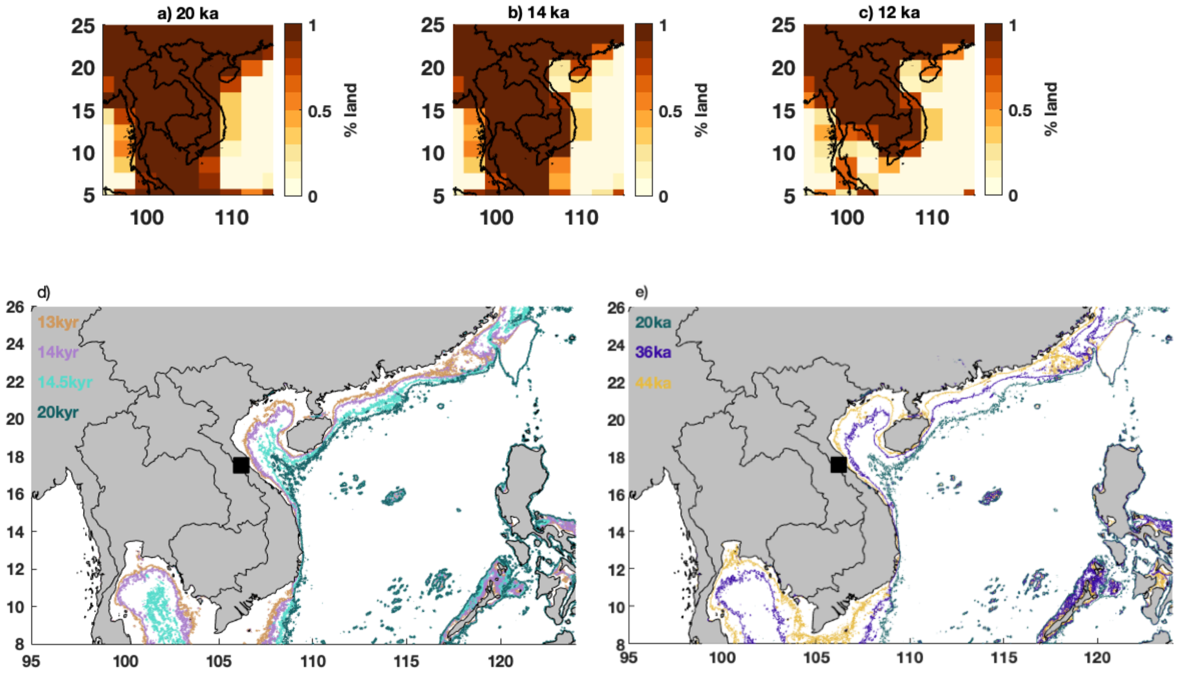


**Fig. S8.** Sea level change during the deglaciation. iTRACE atmosphere model land fraction used at a) 20 ka, b) 14 ka, and c) 12 ka. Each grid cell has a value ranging from 0% to 100% land. Paleoshorelines from the ICE-PC2 global ice history during the d) deglaciation and e) late MIS 3.

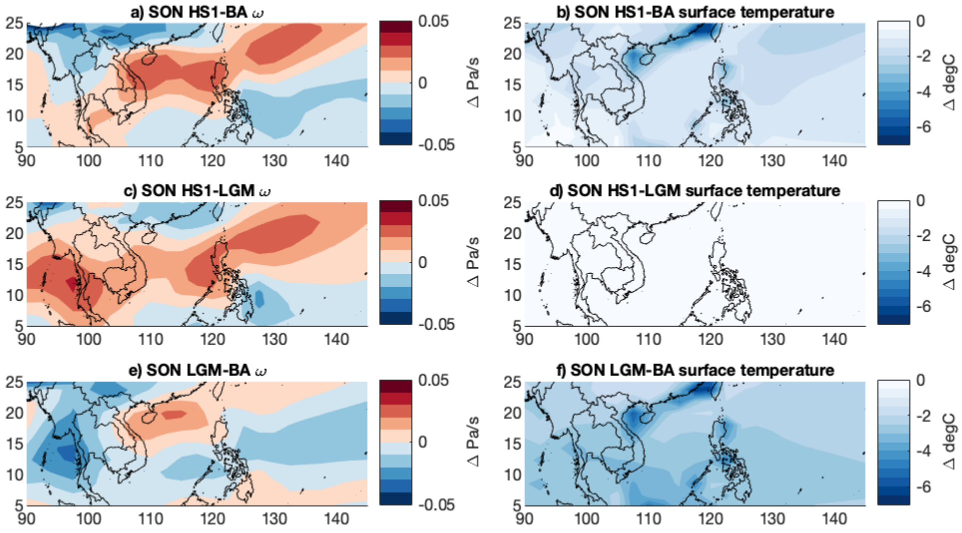


**Fig. S9.** iTRACE JJA vertical velocity ($\omega$) and surface temperature anomalies during for a,b) HS1 – BA, c,d) HS1 – LGM, and e,f) LGM – BA. Note, colors in panel d are muted because temperature change is small.

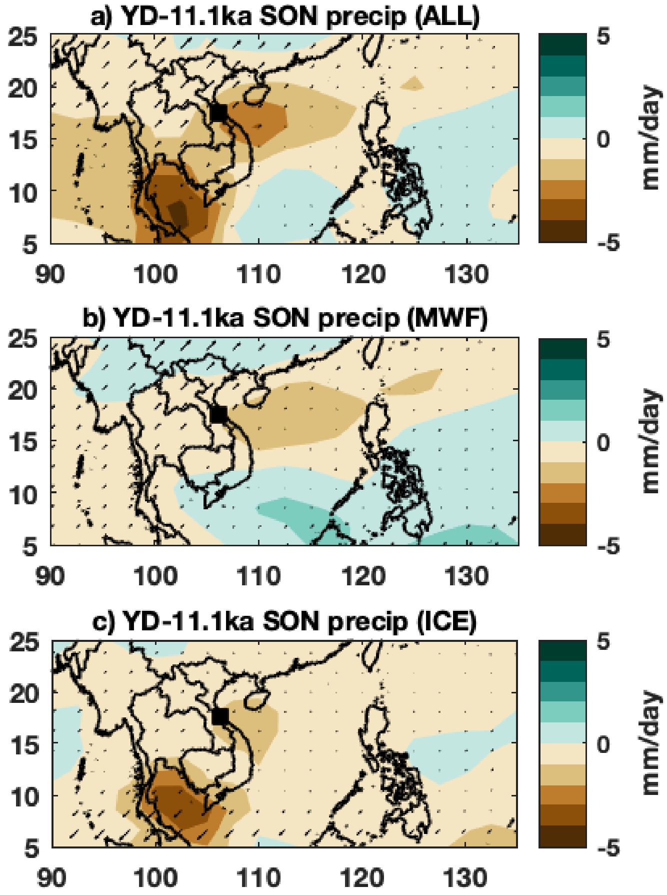


**Fig. S10.** iTRACE precipitation and 850 mbar wind anomalies over MSEA during YD – 11.1 ka for a) all forcings (ALL), b) meltwater forcing (MWF), and c) sea level forcing (ICE). The YD-11.1 ka time period encompasses a period in the iTRACE simulations with enhanced meltwater forcing during the YD and sea level change at 12 ka.

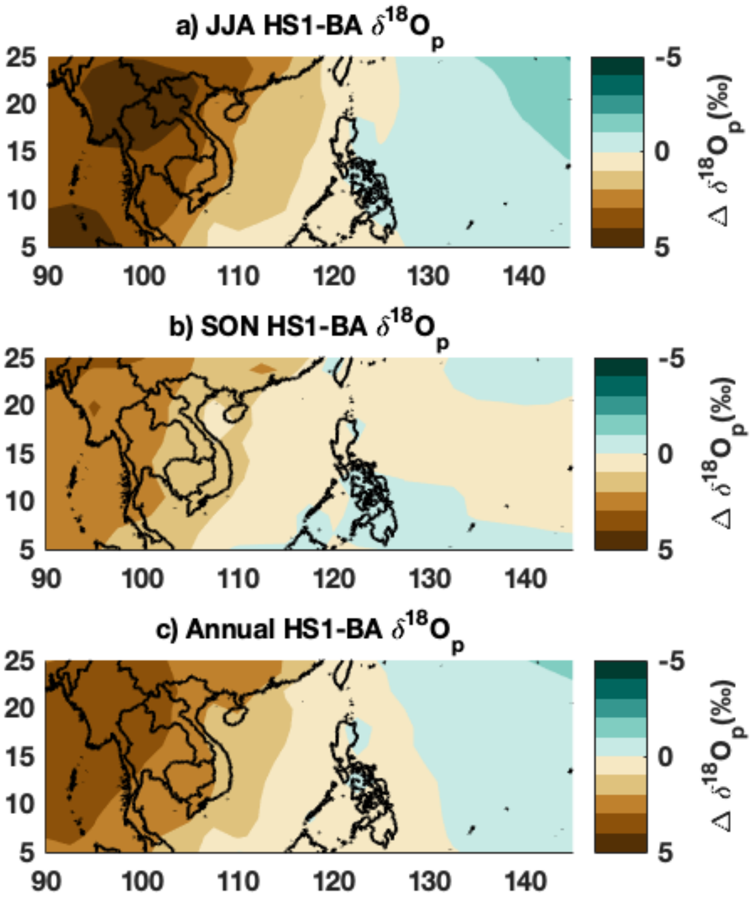


**Fig. S11.** iTRACE a) JJA, b) SON, and c) annual weighted $\delta^{18}O_p$ anomalies during HS1-BA.

**Table S1.** HH-1 U-Th dates.

| Sample ID | Depth (cm) | $^{238}U$ (ng/g) | $^{232}Th$ (pg/g) | δ $^{234}U$ (‰) | $^{230}Th/^{238}U$ (activity) | $^{230}Th/^{232}Th$ (ppm atomic) | $δ^{234}U$ initial (‰) | Raw age (yrs BP) | Corr. age (yrs BP) |
|---|---|---|---|---|---|---|---|---|---|
| UU-H2 | 18.2 | 140 ± 3 | 790 ± 16 | 2728 ± 2 | 0.1444 ± 0.0006 | 405 ± 2 | 2759 ± 2 | 4290 ± 19 | 3961 ± 131 |
| UU-H3 | 44.4 | 82 ± 2 | 388 ± 8 | 2845 ± 1 | 0.1776 ± 0.0008 | 596 ± 3 | 2885 ± 2 | 5128 ± 24 | 4848 ± 107 |
| UU-H4 | 68.7 | 79 ± 2 | 168 ± 3 | 2873 ± 2 | 0.2150 ± 0.0011 | 1599 ± 11 | 2923 ± 2 | 6186 ± 33 | 6022 ± 57 |
| UU-H5 | 95.7 | 87 ± 2 | 180 ± 4 | 2947 ± 1 | 0.2696 ± 0.0013 | 2071 ± 13 | 3011 ± 1 | 7651 ± 37 | 7492 ± 57 |
| HH-U33 | 105.6 | 98 ± 2 | 166 ± 3 | 2897 ± 2 | 0.2828 ± 0.0012 | 2670 ± 12 | 2963 ± 2 | 8144 ± 36 | 8001 ± 51 |
| UU-H6 | 115.6 | 78 ± 2 | 293 ± 6 | 2659 ± 1 | 0.2773 ± 0.0012 | 1167 ± 6 | 2722 ± 2 | 8517 ± 39 | 8272 ± 96 |
| HH-U34 | 132.2 | 94 ± 2 | 571 ± 11 | 2806 ± 2 | 0.3089 ± 0.0014 | 805 ± 4 | 2877 ± 2 | 9140 ± 43 | 8800 ± 142 |
| UU-H7 | 151.8 | 102 ± 2 | 203 ± 4 | 3130 ± 1 | 0.3913 ± 0.0015 | 3128 ± 16 | 3225 ± 2 | 10725 ± 43 | 10574 ± 59 |
| UU-H8 | 180.4 | 101 ± 2 | 457 ± 9 | 2919 ± 2 | 0.4045 ± 0.0014 | 1415 ± 5 | 3016 ± 2 | 11728 ± 43 | 11463 ± 106 |
| UU-H9 | 206.7 | 128 ± 3 | 180 ± 4 | 3146 ± 1 | 0.4603 ± 0.0016 | 5186 ± 31 | 3260 ± 2 | 12652 ± 46 | 12525 ± 54 |
| UU-H10 | 235.9 | 129 ± 3 | 218 ± 4 | 3017 ± 1 | 0.4884 ± 0.0016 | 4570 ± 25 | 3137 ± 1 | 13918 ± 49 | 13777 ± 60 |
| HH1-U25 | 238.0 | 140 ± 3 | 349 ± 7 | 3150 ± 2 | 0.5119 ± 0.0018 | 3248 ± 11 | 3277 ± 2 | 14130 ± 52 | 13958 ± 72 |
| HH1-U37 | 238.3 | 173 ± 3 | 5549 ± 111 | 3666 ± 2 | 0.6238 ± 0.0026 | 308 ± 1 | 3816 ± 7 | 15370 ± 67 | 14148 ± 582 |
| HH-U16 | 238.5 | 115 ± 2 | 1301 ± 26 | 3784 ± 1 | 0.6141 ± 0.0025 | 861 ± 3 | 3941 ± 3 | 14722 ± 63 | 14257 ± 207 |
| HH1-U26 | 239.0 | 128 ± 3 | 1293 ± 26 | 3611 ± 1 | 0.6403 ± 0.0026 | 1004 ± 4 | 3773 ± 3 | 16002 ± 68 | 15566 ± 195 |
| HH1-U27 | 240.5 | 120 ± 2 | 359 ± 7 | 3760 ± 1 | 0.6789 ± 0.0028 | 3595 ± 14 | 3938 ± 2 | 16456 ± 71 | 16280 ± 88 |
| HH-U17 | 242.0 | 112 ± 2 | 468 ± 9 | 4161 ± 2 | 0.8376 ± 0.0033 | 3190 ± 13 | 4387 ± 2 | 18870 ± 79 | 18666 ± 104 |
| HH1-U28 | 242.8 | 112 ± 2 | 572 ± 11 | 3952 ± 2 | 0.8850 ± 0.0033 | 2754 ± 10 | 4190 ± 2 | 20931 ± 85 | 20691 ± 120 |
| HH1-U29 | 243.6 | 115 ± 2 | 361 ± 7 | 4229 ± 2 | 1.0361 ± 0.0035 | 5241 ± 17 | 4517 ± 3 | 23391 ± 86 | 23222 ± 99 |
| HH-U18 | 243.9 | 137 ± 3 | 877 ± 18 | 3857 ± 3 | 1.0180 ± 0.0037 | 2534 ± 9 | 4135 ± 4 | 24882 ± 101 | 24597 ± 147 |
| HH-U19 | 244.9 | 130 ± 3 | 281 ± 6 | 3224 ± 2 | 0.9214 ± 0.0036 | 6803 ± 27 | 3468 ± 2 | 26021 ± 111 | 25868 ± 119 |
| HH-U20 | 254.7 | 131 ± 3 | 508 ± 10 | 3166 ± 2 | 0.9182 ± 0.0031 | 3750 ± 12 | 3408 ± 2 | 26322 ± 98 | 26100 ± 124 |
| HH-U21 | 260.9 | 136 ± 3 | 463 ± 9 | 2835 ± 1 | 0.8750 ± 0.0031 | 4081 ± 14 | 3062 ± 2 | 27362 ± 107 | 27147 ± 129 |
| UU-H11 | 262.4 | 122 ± 2 | 602 ± 12 | 3058 ± 1 | 0.9479 ± 0.0030 | 3056 ± 10 | 3308 ± 2 | 28071 ± 98 | 27804 ± 139 |
| HH1-U30 | 263.8 | 121 ± 2 | 632 ± 13 | 3203 ± 1 | 1.0037 ± 0.0038 | 3042 ± 11 | 3471 ± 2 | 28753 ± 120 | 28481 ± 157 |
| HH-U35 | 264.7 | 99 ± 2 | 827 ± 17 | 3946 ± 2 | 1.2800 ± 0.0040 | 2436 ± 8 | 4308 ± 3 | 31389 ± 110 | 31039 ± 175 |
| HH1-U31 | 265.2 | 86 ± 2 | 386 ± 8 | 3697 ± 2 | 1.2442 ± 0.0044 | 4423 ± 15 | 4048 ± 3 | 32231 ± 129 | 32008 ± 150 |
| HH-U22 | 266.8 | 101 ± 2 | 781 ± 16 | 3290 ± 2 | 1.1746 ± 0.0039 | 2418 ± 7 | 3613 ± 3 | 33492 ± 127 | 33134 ± 192 |
| HH-U23 | 272.0 | 96 ± 2 | 487 ± 10 | 3488 ± 2 | 1.2826 ± 0.0044 | 4008 ± 13 | 3849 ± 3 | 35133 ± 137 | 34882 ± 164 |
| HH-U24 | 280.2 | 121 ± 2 | 99 ± 2 | 3008 ± 2 | 1.2020 ± 0.0045 | 23362 ± 124 | 3341 ± 3 | 37161 ± 162 | 37058 ± 163 |
| HH-U36 | 288.0 | 125 ± 2 | 187 ± 4 | 3014 ± 2 | 1.2210 ± 0.0050 | 12930 ± 50 | 3352 ± 3 | 37771 ± 178 | 37642 ± 181 |
| UU-H12* | 289.8 | 128 ± 3 | 1295 ± 26 | 2997 ± 2 | 1.1988 ± 0.0037 | 1886 ± 5 | 3324 ± 3 | 37173 ± 133 | 36701 ± 241 |
| HH1-U32 | 302.8 | 133 ± 3 | 621 ± 12 | 2870 ± 2 | 1.2136 ± 0.0041 | 4119 ± 13 | 3203 ± 3 | 39140 ± 153 | 38877 ± 180 |
| UU-H13 | 315.9 | 139 ± 3 | 680 ± 14 | 2856 ± 2 | 1.2674 ± 0.0043 | 4120 ± 14 | 3208 ± 3 | 41321 ± 166 | 41050 ± 194 |
| UU-H14 | 348.8 | 135 ± 3 | 672 ± 14 | 2670 ± 2 | 1.2818 ± 0.0039 | 4095 ± 13 | 3025 ± 3 | 44396 ± 162 | 44113 ± 194 |
| UU-H15 | 367.8 | 132 ± 3 | 223 ± 5 | 2660 ± 1 | 1.2991 ± 0.0039 | 12163 ± 59 | 3022 ± 2 | 45252 ± 160 | 45109 ± 164 |

Uncertainties are 2σ. Reported errors for $^{238}U$ and $^{232}Th$ concentrations are estimated to be ±1 % due to uncertainties in spike concentration; analytical uncertainties are smaller. Decay constants for $^{230}Th$ and $^{234}U$ are from (10); decay constant for $^{238}U$ is 1.55125 x $10^{-10}$ $yr^{-1}$ (11). All ages are relative to 1950. Corrected ages assume initial $^{230}Th/^{232}Th$ atomic ratio of 25 ± 12.5 ppm. *Date excluded from age model.

## SI References


1. J. Martín-Chivelet, M. B. Muñoz-García, J. A. Cruz, A. I. Ortega, M. J. Turrero, Speleothem Architectural Analysis: Integrated approach for stalagmite-based paleoclimate research. *Sediment. Geol.* **353**, 28–45 (2017).

2. A. Yatagai, *et al.*, APHRODITE: Constructing a Long-Term Daily Gridded Precipitation Dataset for Asia Based on a Dense Network of Rain Gauges. *Bull. Am. Meteorol. Soc.* **93**, 1401–1415 (2012).

3. E. Kalnay, *et al.*, The NCEP/NCAR 40-Year Reanalysis Project. *Bull. Am. Meteorol. Soc.* **77**, 437–472 (1996).

4. A. F. Stein, *et al.*, NOAA's HYSPLIT atmospheric transport and dispersion modeling system. *Bull. Am. Meteorol. Soc.* **96**, 2059–2077 (2015).

5. A. Wolf, *EasHyClustering: Use Python to plot meteorological data from HYSPLIT clustering* (Github, 2023) https:/doi.org/10.5281/zenodo.7574382 (April 13, 2023).

6. T. Pico, J. R. Creveling, J. X. Mitrovica, Sea-level records from the U.S. mid-Atlantic constrain Laurentide Ice Sheet extent during Marine Isotope Stage 3. *Nat. Commun.* **8**, 15612 (2017).

7. T. Pico, D. McGee, J. Russell, J. X. Mitrovica, Recent constraints on MIS 3 sea level support role of continental shelf exposure as a control on Indo-pacific hydroclimate. *Paleoceanogr. paleoclimatology* **35** (2020).

8. Y. Yokoyama, *et al.*, Rapid glaciation and a two-step sea level plunge into the Last Glacial Maximum. *Nature* **559**, 603–607 (2018).

9. H. Zhang, *et al.*, East Asian hydroclimate modulated by the position of the westerlies during Termination I. *Science* **362**, 580–583 (2018).

10. H. Cheng, *et al.*, Improvements in 230Th dating, 230Th and 234U half-life values, and U–Th isotopic measurements by multi-collector inductively coupled plasma mass spectrometry. *Earth Planet. Sci. Lett.* **371–372**, 82–91 (2013).

11. A. H. Jaffey, K. F. Flynn, L. E. Glendenin, W. C. Bentley, A. M. Essling, Precision measurement of half-lives and specific activities ofU235andU238. *Phys. Rev. C Nucl. Phys.* **4**, 1889–1906 (1971).
